# Alternative 3′ UTR polyadenylation is disrupted in the rNLS8 mouse model of ALS/FTLD

**DOI:** 10.1186/s13041-025-01174-1

**Published:** 2025-01-14

**Authors:** Randall J. Eck, Paul N. Valdmanis, Nicole F. Liachko, Brian C. Kraemer

**Affiliations:** 1 Graduate Program in Neuroscience, University of Washington, Seattle, WA 98195 USA; 2 Division of Gerontology and Geriatric Medicine, Department of Medicine, University of Washington, Seattle, WA 98104 USA; 3 Department of Genome Sciences, University of Washington, Seattle, WA 98195 USA; 4 Division of Medical Genetics, Department of Medicine, University of Washington, Seattle, WA 98195 USA; 5 Geriatrics Research Education and Clinical Center, Veterans Affairs Puget Sound Health Care System, Seattle, WA 98108 USA; 6 Department of Laboratory Medicine and Pathology, University of Washington, Seattle, WA 98195 USA; 7 Department of Psychiatry and Behavioral Sciences, University of Washington, Seattle, WA 98195 USA

**Keywords:** ALS, FTLD, TDP-43, Alternative polyadenylation

## Abstract

Recent research has highlighted widespread dysregulation of alternative polyadenylation in amyotrophic lateral sclerosis (ALS) and frontotemporal lobar degeneration with TDP-43 pathology (FTLD-TDP). Here, we identify significant disruptions to 3` UTR polyadenylation in the ALS/FTLD-TDP mouse model rNLS8 that correlate with changes in gene expression and protein levels through the re-analysis of published RNA sequencing and proteomic data. A subset of these changes are shared with TDP-43 knock-down mice suggesting depletion of endogenous mouse TDP-43 is a contributor to polyadenylation dysfunction in rNLS8 mice. Some conservation exists between alternative polyadenylation in rNLS8 mice and human disease models including in disease relevant genes and biological pathways. Together, these findings support both TDP-43 loss and toxic gain-of-function phenotypes as contributors to the neurodegeneration in rNLS8 mice, nominating its continued utility as a preclinical model for investigating mechanisms of neurodegeneration in ALS/FTLD-TDP.

**Supplementary Information:**

The online version contains supplementary material available at 10.1186/s13041-025-01174-1.

Insoluble inclusions of the RNA binding protein TDP-43 in neurons and glia are pathological hallmarks of several neurodegenerative diseases, including amyotrophic lateral sclerosis (ALS) and frontotemporal lobar degeneration with TDP-43 pathology (FTLD-TDP) [1]. In disease, TDP-43 is most commonly mislocalized into cytoplasmic aggregates and depleted from the nucleus, driving neurodegeneration through both gain-of-function and loss-of-function mechanisms [1]. TDP-43 regulates several aspects of RNA metabolism including RNA splicing and alternative polyadenylation, which recent work has highlighted as relevant to ALS/FTLD-TDP [2–6]. Polyadenylation is the process of adding poly(A) tails to RNA to ensure stability, transport, and translation [7]. Alternative polyadenylation (APA) can occur at several sites within three prime untranslated regions (3` UTR) and intronic regions generating mRNA isoforms with preserved coding regions but varied regulatory elements, affecting RNA stability, localization, and protein isoform expression across diverse tissues and in tissue-specific programs [7]. In ALS/FTLD-TDP, polyadenylation at cryptic APA sites can disrupt coding regions; for example, polyadenylation of a cryptic exon in *STMN2* reduces Stathmin-2 levels contributing to motor neuropathy [8]. This cryptic exon in Stathmin-2 is not conserved in mice [9]. Since disruptions to 3′ UTR APA likely also contribute to RNA dysregulation in disease, we sought to determine if mouse models of ALS/FTLD-TDP display changes to APA.

rNLS8 mice are a widely used transgenic mouse model of ALS/FTLD-TDP that express inducible cytoplasmic localized human TDP-43 (hTDP-43∆NLS) throughout the central nervous system [10]. rNLS8 mice exhibit rapid and progressive motor and behavioral deficits, robust formation of cytoplasmic phosphorylated detergent insoluble TDP-43 aggregates, and substantial neurodegeneration in the brain and spinal cord. Endogenous nuclear mouse TDP-43 is also depleted in rNLS8 mice allowing for the study of both loss and gain-of-function disease mechanisms [1, 10].

Utilizing the computational package APAlyzer [11] (see Additional File 1 for Methods), we identified 217 genes with altered 3′ UTR APA (DEXseq: p adj < 0.05, RED > |0.1|) in the neocortex of rNLS8 mice three weeks post-induction of hTDP-43∆NLS (an early disease stage) compared to controls by reanalyzing RNA sequencing from a study developing TDP-43 targeting peptides [12] (mixed gender, 28 week old, *n* = 27, pair-end reads of 200 bps, read depth of ~ 20,000,000 bps) (Fig. 1A, Table S1, Additional File 1). REPAC [13], an alternative site-specific computational package and database, identified 309 significantly differentially utilized APA sites (p adj < 0.05, cFC > |0.1|) and 199 genes with consistent evidence of 3′ UTR lengthening or shortening between sites, 8 shared with APAlyzer (Fig. 1A, Table S2). We also observed similar 3′ UTR APA changes in an independent nuclear localization signal (NLS) deficient human TDP-43 transgenic mouse which also depletes endogenous mouse TDP-43 (ΔNLS-hTDP-43) (49 significant APA genes shared by APAlyzer and 10 by REPAC with rNLS8) (Table S3 & S4) [14]. Gene ontology analysis by DAVID [15] suggests these polyadenylation changes identified by APAlyzer and REPAC in rNLS8 mice occur in genes found in disease-relevant pathways including cytoplasmic translation, synapse organization, mRNA splicing, and phosphorylation (Fig. 1B, Table S5). Bulk RNA sequencing is limited when evaluating APA and identifying novel or cryptic APA sites as it cannot capture individual cell-specific regulation and is less sensitive than specialized transcriptomic methods, such as 3′-end sequencing [7]. Nevertheless, rNLS8 and ΔNLS-hTDP-43 mice exhibit disruptions to alternative polyadenylation.

**Fig. 1:**
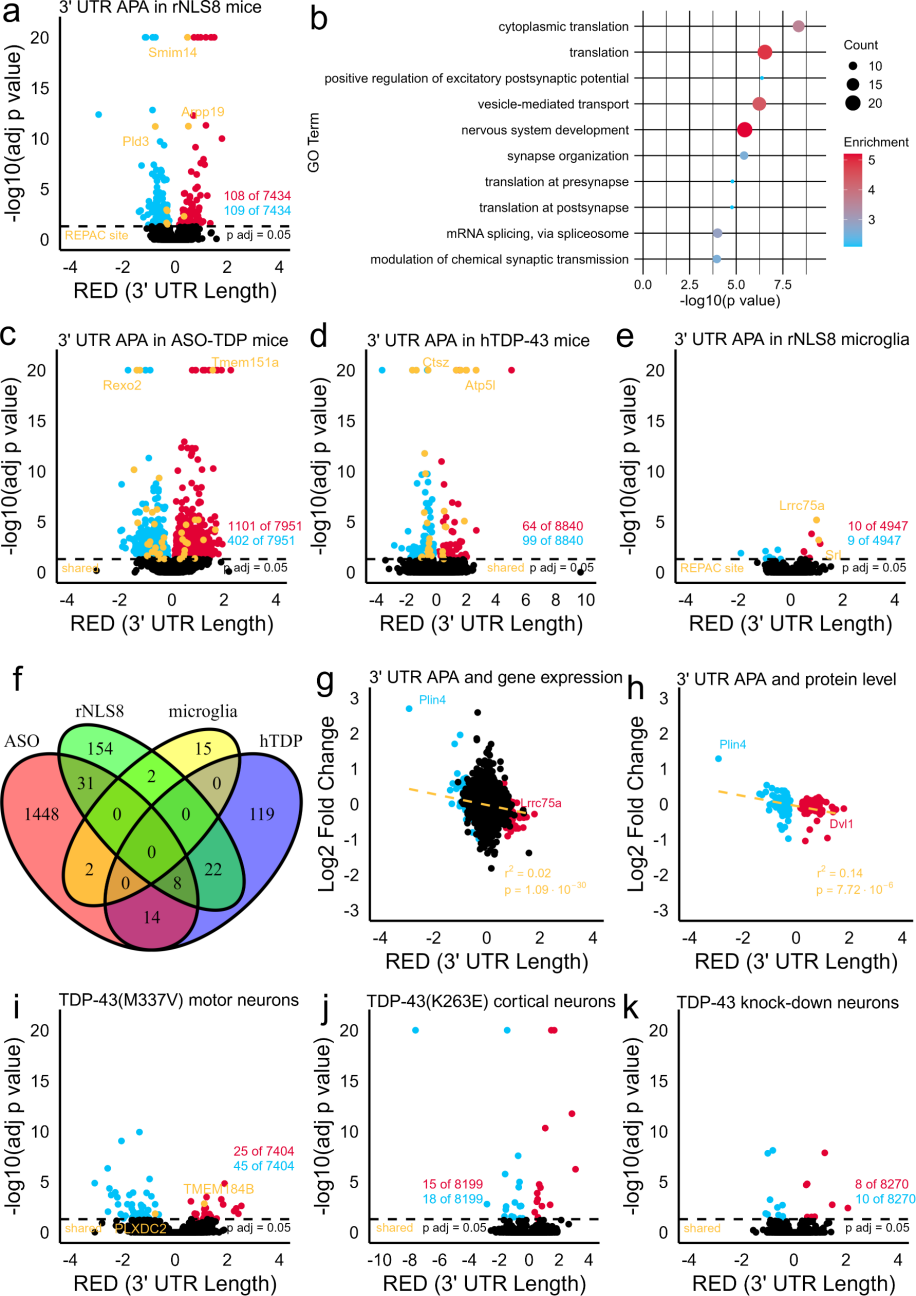
Characterization of alternative polyadenylation in mouse models of ALS/FTLD-TDP. **a** Volcano plot of differential 3′ UTR APA in rNLS8 mice. DEXseq p adjusted values of 0 were set to 1.00e^− 20^ for the purposes of graphing. **b** Dot plot of top 10 enriched biological process GO terms of APA genes in rNLS8 mice. **c** Volcano plot of 3′ UTR APA in non-transgenic mice targeted with a TDP-43 ASO. DEXseq p adjusted values of 0 were set to 1.00e^− 20^ for the purposes of graphing. **d** Volcano plot of 3′ UTR APA in TDP-43 Tg mice. DEXseq p adjusted values of 0 were set to 1.00e^− 20^ for the purposes of graphing. **e** Volcano plot of 3′ UTR APA in rNLS8 mice microglia. **f** Venn diagram of significantly APA genes between the rNLS8, TDP-43 ASO, hTDP-43, and rNLS8 microglia mouse models. **g** Plot of the correlation between relative expression difference of rNLS8 3′ UTR APA and gene expression. **h** Plot of the correlation between relative expression difference of significant rNLS8 3′ UTR APA and protein level changes in an rNLS8 proteomic database four weeks post-transgene induction. **i** Volcano plot of 3′ UTR APA in TDP-43(M337V) iPSC motor neurons. **j** Volcano plot of 3′ UTR APA in TDP-43(K263E) iPSC cortical neurons. DEXseq p adjusted values of 0 were set to 1.00e^− 20^ for the purposes of graphing. **k** Volcano plot of 3′ UTR APA in TDP-43 knock-down in human motors neurons

To evaluate the contribution of TDP-43 loss and gain-of-function mechanisms to these polyadenylation changes, we first compared polyadenylation changes in rNLS8 mice with those in the striatum of non-transgenic C57BL/6J mice depleted of endogenous TDP-43 with anti-sense oligonucleotides (ASOs) from [16] with APAlyzer and REPAC (female, 8–10 week old, *n* = 21, single end reads of 72 bps, read depth ~ 20,000,000 bps) (Fig. 1C, Table S6 & S7). Of the 217 genes with significant differential 3′ UTR APA identified by APAlyzer in rNLS8 mice, 39 were shared in TDP-43 ASO mice (19 were shared by REPAC). TDP-43 ASO mice differentially APA 1,503 3` UTRs by APAlyzer suggesting a dose-dependent relationship between TDP-43 depletion and 3` UTR APA. Second, we utilized CLIP-seq data of mouse TDP-43 to identify polyadenylation changes in genes known to be targeted by TDP-43 [16]. Mouse TDP-43 binds 75.6% of significant APA genes at one or more sites. 6.5% of significant APA genes have direct evidence of TDP-43 3′ UTR binding (Table S8). Third, we compared rNLS8 APA with changes in the spinal cord of transgenic mice expressing wildtype human TDP-43 under the Thy1.2 promoter [17] to evaluate the contribution of gain-of-function mechanisms to APA (male, 6 week old, *n* = 4, pair end read length 200 bps, read depth ~ 75,000,000 bps). We found that rNLS8 mice share 30 differential APA 3′ UTRs with TDP-43 transgenic (Tg) mice by APAlyzer (8 shared by REPAC) (Fig. 1D, Table S9 & S10). Lastly, we compared rNLS8 APA with changes found in primary cortical microglia isolated from rNLS8 mice two weeks post-transgene induction from [18] to evaluate the contribution of non-neuronal cell types to the APA we observed in whole rNLS8 brains (mixed gender, > 20 week old, *n* = 21, pair end reads of 100 bps, read depth ~ 5,000,000 bps). We found that rNLS8 mice microglia display many fewer changes in 3′ UTR APA compared to whole rNLS8 brains, sharing only 2 APA 3′ UTRs by APAlyzer, likely due to the absence of TDP-43 pathology in rNLS8 mice microglia (5 shared by REPAC) (Fig. 1E, Table S11 & S12). Taken together, these results suggest that both depletion of endogenous mouse TDP-43 from the nucleus and toxic transgenic TDP-43 in neurons are contributors to polyadenylation changes in rNLS8 mice (Fig. 1F).

APA can change 3′ UTR length to influence the localization and stability of mRNA transcripts, with longer 3′ UTRs associated with reduced gene expression thanks to additional microRNA and RNA binding protein binding sites [7]. To determine the functional relevance of 3′ UTR APA in rNLS8 mice, we examined the contribution of APA to differential gene expression. Changes in 3′ UTR length in rNLS8 mice explain approximately 2% (r^2^ = 0.02, *p* = 1.09e^− 30^) of differential gene expression in all APA genes examined, with longer 3′ UTRs associated with reduced gene expression (Fig. 1G, Table S13). When just considering those genes with significant APA, approximately 38% (r^2^ = 0.38, *p* = 3.29e^− 24^) of differential gene expression can be linked to changes in 3′ UTR length (Fig. 1G, Table S13). As gene expression is only a proxy for alterations in protein levels, we examined the protein levels of significant APA genes in a proteomic database of 14-week-old rNLS8 mice four weeks post-transgene induction representing an early stage of disease [19]. We found that 55 of the 217 genes with altered 3′ UTR APA were significantly differentially abundant (*p* < 0.05), with the lengthening or shortening of 3′ UTR correlated (r^2^ = 0.14, *p* = 7.72e^− 6^) with changes in protein levels in the 137 significant APA genes for which proteomics data exists (Fig. 1H, Table S14). Altered 3` UTR APA poorly correlated with protein levels for these genes at one week (r^2^ = 0.03) and two weeks post-transgene induction (r^2^ = 0.04), likely because 3` UTR APA was measured three weeks post-transgene induction (Table S14). This relationship was strengthened for protein levels measured at six weeks post-transgene induction (r^2^ = 0.18) and subsequently decreased following a two week recovery period without transgene expression (r^2^ = 0.14) (Table S14). Overall, these data support a progression of and functional impact for polyadenylation changes in rNLS8, but additional experimental validation is required to draw conclusive claims.

Lastly, we compared APA in rNLS8 mice to APA in human cell culture models of ALS. Familial ALS (fALS) patient-derived motor neurons with mutant TDP-43(M337V) from [20] significantly differentially alternatively polyadenylate the 3′ UTRs of 70 genes compared to healthy, age-matched controls (*n* = 5, *N* = 10, pair-end reads of 300 bps, read depth of ~ 10,000,000 bps) (Fig. 1I, Table S15). Of the 217 genes significantly APA in rNLS8 mice by APAlyzer, only 2 have human homologs with APA changes shared in TDP-43(M337V) motor neurons. By REPAC, 2 of 199 mouse genes have human homologs with similar significant APA site usage differences in TDP-43(M337V) motor neurons (Table S16). In fALS TDP-43(K263E) iPSC-derived cortical neurons, no significant APA genes by APAlyzer and 1 gene by REPAC are shared with rNLS8 mice [21] (Fig. 1J, Table S17 & S18) (*n* = 6, pair-ends reads of 300 bps, read depth of ~ 25,000,000 bps). rNLS8 mice and human motor neurons with knock-down of TDP-43 with RNAi [22] share no homologous significant APA genes by APAlyzer and 3 genes by REPAC (Fig. 1K, Table S19 & S20) (*n* = 12, pair-end reads of 150 bps, read depth of ~ 20,000,000 bps). Finally, we compared APA genes in rNLS8 mice from APAlyzer with a list of published genes with altered APA in TDP-43 knock-down iNeurons by 3′-end sequencing, a more specialized transcriptomic method [3]. 18 APA genes in rNLS8 were shared (Table S21). Altogether, 23 genes or approximately 10% of significantly APA 3′ UTRs identified by APAlyzer in rNLS8 mice are also significantly and similarly disrupted in at least one human cell culture model of ALS (Table S22). These include disease-relevant genes (e.g. *Usp14* and *Rita1*) as well as genes in disease relevant pathways (*Dnaja1*: chaperone-mediate protein folding and *Tmem184b*: synaptic activity) [23–26].

In summary, rNLS8 Tg mice exhibit ALS/FTLD-TDP features including both toxic gain-of-function and TDP-43 loss-of-function phenotypes, including the accumulation of phosphorylated TDP-43 containing lesions (the most consistent neuropathology of ALS/FTLD-TDP) [10, 27] and loss-of-function changes in RNA regulation related to nuclear depletion of TDP-43. Here, we identify dysregulation of 3′ UTR APA in rNLS8 mice which correlate with gene expression and protein level changes, have some conservation with human disease models, and show signatures of a TDP-43 loss-of-function mechanism, providing additional evidence endogenous nuclear TDP-43 activity is reduced in rNLS8 mice (Table S23). Taken together, the evidence for both TDP-43 loss-of-nuclear function and toxic gain-of-cytoplasmic function suggest the continued use of rNLS8 mice as a robust model for preclinical testing of interventions for ALS/FTLD-TDP.

## Electronic supplementary material

Below is the link to the electronic supplementary material.

Supplementary Material 1: Additional file 1: Methods

Supplementary Material 2: Additional file 2. **Table S1.** APAlyzer analysis of rNLS8 mice. **Table S2.** REPAC analysis of rNLS8 mice. **Table S3.** APAlyzer analysis of TDP-43ΔNLS mice. **Table S4.** REPAC analysis of TDP-43ΔNLS mice. **Table S5.** DAVID analysis of APA genes in rNLS8 mice. **Table S6**. APAlyzer analysis of mice targeted with TDP-43 ASOs. **Table S7.** REPAC analysis mice targeted with TDP-43 ASOs. **Table S8.** CLIP-seq analysis of mouse TDP-43 binding sites. **Table S9.** APAlyzer analysis of TDP-43 Tg mice. **Table S10.** REPAC analysis of TDP-43 Tg mice. **Table S11.** APAlyzer analysis of microglia from rNLS8 mice. **Table S12.** REPAC analysis of microglia from rNLS8 mice. **Table S13.** Gene expression of APA genes in rNLS8 mice. **Table S14.** Protein levels of APA genes from a proteomic database. **Table S15.** APAlyzer analysis of TDP-43(M337V) iPSC motor neurons. **Table S16.** REPAC analysis of TDP-43(M337V) iPSC motor neurons. **Table S17.** APAlyzer analysis of TDP-43(K263E) iPSC cortical neurons. **Table S18.** REPAC analysis of TDP-43(K263E) iPSC cortical neurons. **Table S19.** APAlyzer analysis of TDP-43 RNAi knock-down in human motor neurons. **Table S20.** REPAC analysis of TDP-43 RNAi knock-down in human motor neurons. **Table S21.** A comparison of APA genes identified by APAlyzer in rNLS8 mice with list of APA genes in iNeurons from 3′-end sequencing. **Table S22.** A list of APA genes identified by APAlyzer in rNLS8 mice with evidence of conservation in human disease models. **Table S23.** A table summarizing the methodological details and results for each set of RNA sequencing data utilized

## Data Availability

All data analyzed during this study are included in this published article and its supplementary information files.

## Abbreviations

ALS: Amyotrophic lateral sclerosis
FTLD-TDP: Frontotemporal lobar degeneration with TDP-43 pathology
TDP-43: TAR DNA-binding protein 43
APA: Alternative polyadenylation
3′UTR: Three prime untranslated region
NLS: Nuclear localization signal
RED: Relative expression difference
bps: Base pairs
cFC: Change in log fold change
Tg: Transgenic
ASOs: Anti-sense oligonucleotides
CLIP-seq: Cross-linking and immunoprecipitation sequencing
