# Supplementary material for: Alternative 3′ UTR polyadenylation is disrupted in the rNLS8 mouse model of ALS/FTLD
Source: Mol Brain. 2025 Jan 14;18:1. doi: 10.1186/s13041-025-01174-1 (PMC11734424; doi:10.1186/s13041-025-01174-1)
Supplement: Supplementary file 1 — Supplementary Material 1: Additional file 1: Methods [file 13041_2025_1174_MOESM1_ESM.docx]

**Methods**

**RNA Sequencing**

Raw RNA sequencing fastq files were downloaded from NCBI GEO (1) GSE233669 (2),  GSE65973 (3), GSE27394 (4), GSE147544 (5), GSE196144 (6), and GSE121569 (7) as well as PRJNA1104758 (8) and PRJNA624791 (9) respectively.

**RNA Sequencing Alignment**

RNA sequencing reads were mapped to the mouse genome mm10 (10) or human genome hg38 (10) with STAR (version 2.7.11a) (11) using the public server at usegalaxy.org (version 24) and default parameters (12).

**Alternative Polyadenylation Analysis**

APAlyzer (version 1.18.0) (13) via Bioconductor (14) in R (version 4.4.0) (15) was used to identify alternative polyadenylation of three prime untranslated regions (3’ UTR APA). The default APAlyzer reference of 3’ UTR APA in the mouse genome mm10 and human genome hg38 generated from PolyA_DB databases (16) were used. APAlyzer relies upon reads aligning to the regions between the proximal polyadenylation site and the gene stop codon and those aligning to the region between the distal and proximal polyadenylation sites, allowing for the capture of some cryptic APA driven changes in 3’ UTR length. Relative expression difference (RED) and significance for 3’ UTR regions were calculated by APAlyzer (10) and by DEXseq (version 1.50.0) (17) with default settings. DEXseq p adjusted values of 0 were set to 1.00e^-20^ for the purposes of graphing. Only DEXseq values for genes found also in the APAlyzer analysis output are displayed. An increase in RED denotes 3’UTR lengthening, and a decrease denotes 3’UTR shortening (13). APAlyzer with DEXseq has been used to study 3’ UTR APA in TDP-43 proteinopathies before (18). REPAC (version 0.99.0) in R (version 4.4.0) (15) was used to identify site-specific 3’ UTR APA. The default REPAC reference of 3’ UTR APA sites in the mm10 and hg38 genomes generated from PolyAsite databases (19) was used. We selected REPAC to compliment APAlyzer because each utilize two distinct approaches and databases to quantifying APA . REPAC utilizes reads aligning 50 base pairs upstream of a polyadenylation site to calculate significance and change in log fold site usage. Default settings were used. Visualization made with ggplot2 and VennDiagram (20).

**Gene Enrichment Analysis**

DAVID (version v2024q2) functional annotation bioinformatic analysis was used to determine gene ontology over-representation of biological processes for APA genes (21).

**CLIP-Seq Analysis**

CLIP-seq reads from SRR107032 (4) were aligned to the mouse genome mm10 (10) with STAR (version 2.7.11a) (11) using the public server at usegalaxy.org (version 24.1) (12). MACS2 (version 2.2.9.1) (22) was used to call CLIP-seq peaks and ChIPSeeker (version 1.28.3) (23) was used to annotate peaks (12).

**Differential Gene Expression Analysis**

Gene counts were generated with featureCounts (version 2.0.3) (24) using the public server at usegalaxy.org (version 24.1) (12) counting pair-end reads as a single fragment and default parameters. DESeq2 (version 2.11.40.8) (25) was used to determine differentially expressed genes using the public server at usegalaxy.org (version 24.1) and default parameters (12).

**Additional References**

1. Clough E, Barrett T, Wilhite SE, Ledoux P, Evangelista C, Kim IF, et al. NCBI GEO: archive for gene expression and epigenomics data sets: 23-year update. Nucleic Acids Res. 2024;52(D1):D138-D44.

2. Riemenschneider H, Simonetti F, Sheth U, Katona E, Roth S, Hutten S, et al. Targeting the glycine-rich domain of TDP-43 with antibodies prevents its aggregation in vitro and reduces neurofilament levels in vivo. Acta Neuropathol Commun. 2023;11(1):112.

3. Amlie-Wolf A, Ryvkin P, Tong R, Dragomir I, Suh E, Xu Y, et al. Transcriptomic Changes Due to Cytoplasmic TDP-43 Expression Reveal Dysregulation of Histone Transcripts and Nuclear Chromatin. PLoS One. 2015;10(10):e0141836.

4. Polymenidou M, Lagier-Tourenne C, Hutt KR, Huelga SC, Moran J, Liang TY, et al. Long pre-mRNA depletion and RNA missplicing contribute to neuronal vulnerability from loss of TDP-43. Nat Neurosci. 2011;14(4):459-68.

5. Dafinca R, Barbagallo P, Farrimond L, Candalija A, Scaber J, Ababneh NA, et al. Impairment of Mitochondrial Calcium Buffering Links Mutations in C9ORF72 and TARDBP in iPS-Derived Motor Neurons from Patients with ALS/FTD. Stem Cell Reports. 2020;14(5):892-908.

6. Imaizumi K, Ideno H, Sato T, Morimoto S, Okano H. Pathogenic Mutation of TDP-43 Impairs RNA Processing in a Cell Type-Specific Manner: Implications for the Pathogenesis of ALS/FTLD. eNeuro. 2022;9(3).

7. Klim JR, Williams LA, Limone F, Guerra San Juan I, Davis-Dusenbery BN, Mordes DA, et al. ALS-implicated protein TDP-43 sustains levels of STMN2, a mediator of motor neuron growth and repair. Nat Neurosci. 2019;22(2):167-79.

8. Carmen-Orozco RP, Tsao W, Ye Y, Sinha IR, Chang K, Trinh VT, et al. Elevated nuclear TDP-43 induces constitutive exon skipping. Mol Neurodegener. 2024;19(1):45.

9. Hunter M, Spiller KJ, Dominique MA, Xu H, Hunter FW, Fang TC, et al. Microglial transcriptome analysis in the rNLS8 mouse model of TDP-43 proteinopathy reveals discrete expression profiles associated with neurodegenerative progression and recovery. Acta Neuropathol Commun. 2021;9(1):140.

10. Raney BJ, Barber GP, Benet-Pagès A, Casper J, Clawson H, Cline MS, et al. The UCSC Genome Browser database: 2024 update. Nucleic Acids Res. 2024;52(D1):D1082-D8.

11. Dobin A, Davis CA, Schlesinger F, Drenkow J, Zaleski C, Jha S, et al. STAR: ultrafast universal RNA-seq aligner. Bioinformatics. 2013;29(1):15-21.

12. Community G. The Galaxy platform for accessible, reproducible and collaborative biomedical analyses: 2022 update. Nucleic Acids Res. 2022;50(W1):W345-W51.

13. Wang R, Tian B. APAlyzer: a bioinformatics package for analysis of alternative polyadenylation isoforms. Bioinformatics. 2020;36(12):3907-9.

14. Gentleman RC, Carey VJ, Bates DM, Bolstad B, Dettling M, Dudoit S, et al. Bioconductor: open software development for computational biology and bioinformatics. Genome Biol. 2004;5(10):R80.

15. Team RC. R: A language and environment for statistical computing. R Foundation for Statistical Computing, Vienna, Austria. <https://www.R-project.org/2021>.

16. Wang R, Nambiar R, Zheng D, Tian B. PolyA_DB 3 catalogs cleavage and polyadenylation sites identified by deep sequencing in multiple genomes. Nucleic Acids Res. 2018;46(D1):D315-D9.

17. Anders S, Reyes A, Huber W. Detecting differential usage of exons from RNA-seq data. Genome Res. 2012;22(10):2008-17.

18. Zeng Y, Lovchykova A, Akiyama T, Liu C, Guo C, Jawahar VM, et al. TDP-43 nuclear loss in FTD/ALS causes widespread alternative polyadenylation changes. bioRxiv. 2024.

19. Herrmann CJ, Schmidt R, Kanitz A, Artimo P, Gruber AJ, Zavolan M. PolyASite 2.0: a consolidated atlas of polyadenylation sites from 3' end sequencing. Nucleic Acids Res. 2020;48(D1):D174-D9.

20. Chen H, Boutros PC. VennDiagram: a package for the generation of highly-customizable Venn and Euler diagrams in R. BMC Bioinformatics. 2011;12:35.

21. Sherman BT, Hao M, Qiu J, Jiao X, Baseler MW, Lane HC, et al. DAVID: a web server for functional enrichment analysis and functional annotation of gene lists (2021 update). Nucleic Acids Res. 2022;50(W1):W216-W21.

22. Zhang Y, Liu T, Meyer CA, Eeckhoute J, Johnson DS, Bernstein BE, et al. Model-based analysis of ChIP-Seq (MACS). Genome Biol. 2008;9(9):R137.

23. Yu G, Wang LG, He QY. ChIPseeker: an R/Bioconductor package for ChIP peak annotation, comparison and visualization. Bioinformatics. 2015;31(14):2382-3.

24. Liao Y, Smyth GK, Shi W. featureCounts: an efficient general purpose program for assigning sequence reads to genomic features. Bioinformatics. 2014;30(7):923-30.

25. Love MI, Huber W, Anders S. Moderated estimation of fold change and dispersion for RNA-seq data with DESeq2. Genome Biol. 2014;15(12):550.
